# Reference-free k-mer based dissimilarity measures for metagenomes comparison

**DOI:** 10.3389/fbinf.2026.1788907

**Published:** 2026-05-19

**Authors:** Giorgio Gallina, Cinzia Pizzi

**Affiliations:** Department of Information Engineering, University of Padova, Padova, Italy

**Keywords:** alignment-free, Bray-Curtis, correlation, dissimilarity measures, Jaccard, k-mers, metagenomics samples comparison, reference-free

## Abstract

**Motivation:**

Metagenomics plays a crucial role in unraveling the relationship between microbial communities and the environment in which they live, allowing the development of food and environmental control techniques. Similarly, the study of microbial environments within the human body plays a crucial role towards precision medicine. In these contexts, the problem of metagenomic samples comparison is among the most challenging from the computational point of view due to the size of the datasets and to the incompleteness of microbial databases. Thus, the ability to define and efficiently compute reference-free dissimilarity measures is key to the development of effective and practical tools for metagenomes comparison.

**Results:**

In this work, we present a systematic experimental validation of reference-free k -mer–based dissimilarity measures. To this purpose, we investigate the correlation between two popular ecological dissimilarity measures, Bray-Curtis and Jaccard, computed using reference-free and reference-based k -mer approaches, for 12≤k≤31 . Our experiments cover both simulated and real metagenomics settings (samples from the human body and the oceans), and consider both linear and ranking correlation between the computed values. Our results support the hypothesis that the two definitions are indeed correlated for a wide range of values of k , and promote the development of efficient reference-free computational tools based on k -mer statistics for metagenomes comparison.

## 1 Introduction

Metagenomics, i.e., the analysis of environmental genetic material, is increasingly popular for inferring essential information about microbial life and its interaction with ecosystems for environmental control and food safety [e.g., (Zhang et al., 2021; Billington et al., 2022; Kovac, 2019)]. Similarly, the study of microbial environments within the human body is providing new insights about our microbiome, paving the way towards precision medicine (The Human Microbiome Project Consortium, 2012; Ogunrinola et al., 2020).

The development of computational tools for the analysis of metagenomes have successfully supported several areas of investigations, such as the taxonomic classification of reads from metagenomics samples (Ounit et al., 2015; Lu et al., 2022; Girotto et al., 2017a; Liang et al., 2020; Kim and Steinegger, 2024), metagenomics reads binning (Andreace et al., 2021; Wu and Ye, 2011; Girotto et al., 2017b; Wang et al., 2012; Tomasella and Pizzi, 2025), and metagenomic contigs binning (Mallawaarachchi et al., 2020; Líndez et al., 2023; Wang et al., 2024).

In this study we focus on the problem of the comparison of metagenomic samples, i.e., the assessment of biotic dissimilarity between microbial environments. In recent years, metagenomes comparison has grown attention from the research community not only for its biological implications, but also for its computational challenges (Comin et al., 2020). In fact, current sequencing technologies allow us to produce Terabytes of metagenomic data with little effort, and the alignment-based comparison of datasets of such size requires a large amount of computational resources. In addition to the computational costs, reference-based approaches (either alignment-based or alignment-free) also have limitations: they require curated databases and their results are biased by database incompleteness. For these reasons, approaches that are both reference- and alignment-free are particular appealing to overcome both incompleteness and scalability issues, to successfully tackle the problem of metagenomes comparison.

The first computational efforts to obtain efficient tools for the comparison of metagenomics samples, modeled as sets of reads, only considered the shared content in terms of the presence/absence of reads or k -mers (e.g., (Maillet et al., 2012; Nicolas et al., 2014; Ondov et al., 2016)). Extensions to a variety of more powerful environmental distances were the subject of the seminal paper (Dubinkina et al., 2016). In that paper, the definition of dissimilarity measures (in terms of beta diversity) was proposed for the first time completely based on the k mer content of the samples, also showing high correlation with respect to the results computed using reference-based approaches. Based on these results, several alignment-free and reference-free algorithms have been proposed in recent years for the comparison of metagenomics samples (Ulyantsev et al., 2016; Choi et al., 2018; Benoit et al., 2016; Pellegrina et al., 2020; Rowe et al., 2019). However, in (Dubinkina et al., 2016) the study was limited to k<=11 (and on gut metagenomics only), and even more recent investigations focused on small values of k (k≤5) , simulated data, and tools comparisons (Zhai and Fukuyama, 2023; Ponsero et al., 2023). In this work, to complement previous studies, we designed a set of experiments to assess the correlation between dissimilarity measures computed using reference-free k -mer-based approaches, and reference-based approaches, for large values of k and in different kinds of real environments. For this purpose, we considered two popular measures of comparison among metagenomics samples: the frequency-based Bray-Curtis dissimilarity measure and the presence-based Jaccard distance. The results on simulated and real metagenomics datasets, collected from the environment and the human body, show that the correlation hypothesis holds for large values of k according to both values-based and rank-based measures of correlations, thus validating and promoting the use of k -mer–based definitions of dissimilarities as reliable alternatives for determining sample distances when reference-based profiling is infeasible or biased.

## 2 Materials and methods

The evaluation of the power of k -mer based definitions of dissimilarity measures among metagenomics samples has been carried out under several conditions, which are summarized here and explained in detail in the following subsections:

- We analyzed datasets of both simulated and real metagenomics samples from the natural environment and from the human body;
- We considered both an abundance-based index (Bray-Curtis) and a presence-based index (Jaccard);
- We considered several ranges of lengths k of the k -mers, depending on the size of the dataset to analyze;
- We assessed the correlation of k -mer-based alignement free dissimilarity measures with respect to the ground truth for a simulated dataset, and with respect to a reference-based approach for real datasets;
- We used both the Pearson’s and Spearman’s correlation under the Mantel test with 999 iterations;
- All of our investigations were carried out at the species level.

### 2.1 Datasets

#### 2.1.1 The CAMI II toy human metagenome project dataset

Common practice in empirical studies is to compare results with known ground truth to establish their trustworthiness. For this purpose, we applied our analysis pipeline to a dataset of 12 synthetic WGS short-read metagenomes provided by the CAMI initiative (Meyer et al., 2022) simulating Human Metagenome Project (HMP) samples. These metagenomes are approximately equal in size and all of their paired-end reads are 150 bp long and can be retrieved at https://cami-challenge.org/datasets/ToyHumanMicrobiomeProject/. In the dataset there are three samples for each collection site (Airways, Gastrointestinal Tract, Oral Cavity and Skin). The details of this dataset are reported in Table 1.

**TABLE 1:** CAMI Dataset details. In each dataset the number of reads is $3.333\times 10^{7}$ and the average read length 150bp.

Label	Sample name	Body site
A04	CAMI_airways4	Airways
A07	CAMI_airways7	Airways
A08	CAMI_airways8	Airways
G00	CAMI_gastro0	Gastrointestinal tract
G01	CAMI_gastro1	Gastrointestinal tract
G02	CAMI_gastro2	Gastrointestinal tract
O06	CAMI_oral6	Oral cavity
O07	CAMI_oral7	Oral cavity
O08	CAMI_oral8	Oral cavity
S01	CAMI_skin1	Skin
S13	CAMI_skin13	Skin
S14	CAMI_skin14	Skin

#### 2.1.2 The human metagenome project (HMP) dataset

We downloaded a collection of 12 metagenomes from two different body sites of healthy humans: gastrointestinal tract (stools) and oral cavity (supra-gingival plaque, tongue dorsum) from the web page www.hmpdacc.org/hmp/HMASM/#data of the HMP^1^. These datasets model a collection of large metagenomes of comparable size (about $10^{8}$ reads each). These samples were collected as 101bp paired-end reads on the Illumina GAIIx platform. Human sequences and duplicated reads resulting as artifacts of the sequencing technology were removed, and low quality sequences were trimmed. Reads for which their paired-end mate had been excluded by quality filtering were also discarded (The Human Microbiome Consortium, 2012). For a detailed description of the characteristics of the data sets, we refer to Table 2.

**TABLE 2:** HMP Dataset details. The read number per dataset is about $10^{8}$ and the read length is about 100bp for all datasets.

Label	Sample	Body site	Sampling site
SGP1	SRS053917	Oral cavity	Supragingival plaque
SGP2	SRS075410	Oral cavity	Supragingival plaque
SGP3	SRS011126	Oral cavity	Supragingival plaque
ST1	SRS012273	Gastrointestinal tract	Stool
ST2	SRS045713	Gastrointestinal tract	Stool
ST3	SRS016095	Gastrointestinal tract	Stool
ST4	SRS011239	Gastrointestinal tract	Stool
ST5	SRS024388	Gastrointestinal tract	Stool
TD1	SRS012279	Oral cavity	Tongue dorsum
TD2	SRS011306	Oral cavity	Tongue dorsum
TD3	SRS023617	Oral cavity	Tongue dorsum
TD4	SRS062761	Oral cavity	Tongue dorsum

#### 2.1.3 The global ocean sampling expedition (GOS) dataset

A popular metagenomics dataset used for benchmarking consists of small oceanic metagenomes collected by the Global Ocean Sampling Expedition. Of these we considered the 37 samples analyzed in (Rusch et al., 2007). These samples are rather heterogeneous in size, with up to one order of magnitude of difference, and are composed of sequences of average length about 1070 bp. See Table 3 for further details on the provenance of the samples and for the reference classification obtained by (Rusch et al., 2007) on a fine and broad scale.

**TABLE 3:** GOS Dataset details. The average read length in a dataset is between 1035.97bp and 1106.01bp.

Label	Sample name	Reads $\times 10^{5}$	Rusch fine category	Rusch broad category	Geographic provenience
E01	GOS011	1.244	Estuary	Temperate	Estuary NAEC
E02	GOS012	1.262	Estuary	Temperate	Estuary NAEC
NC1	GOS020	2.964	Not Classified	Not Classified	Freshwater Panama
NC2	GOS025	1.207	Not Classified	Not Classified	Trop. East Pacific
NC3	GOS032	1.480	Not class	Not class	Galapagos
NC4	GOS033	6.923	Not class	Not class	Galapagos
TG01	GOS014	1.289	Galapagos	Tropical	Gulf stream
TG02	GOS021	1.318	Galapagos	Tropical	Trop. East Pacific
TG03	GOS022	1.217	Galapagos	Tropical	Trop. East Pacific
TG04	GOS027	2.221	Galapagos	Tropical	Galapagos
TG05	GOS028	1.891	Galapagos	Tropical	Galapagos
TG06	GOS029	1.315	Galapagos	Tropical	Galapagos
TG07	GOS030	3.592	Galapagos	Tropical	Galapagos
TG08	GOS031	4.364	Galapagos	Tropical	Galapagos
TG11	GOS034	1.343	Galapagos	Tropical	Galapagos
TG12	GOS035	1.408	Galapagos	Tropical	Galapagos
TG13	GOS036	0.775	Galapagos	Tropical	Galapagos
TG14	GOS037	0.657	Galapagos	Tropical	Trop. East Pacific
TG15	GOS047	0.660	Galapagos	Tropical	Trop. South Pacific
TG16	GOS051	1.290	Galapagos	Tropical	Polynesia Archipelagos
TN1	GOS002	1.216	Temp. North	Temperate	Coast NAEC
TN2	GOS003	0.616	Temp. North	Temperate	Coast NAEC
TN3	GOS004	0.530	Temp. North	Temperate	Coast NAEC
TN4	GOS005	0.611	Temp. North	Temperate	Embayment NAEC
TN5	GOS006	0.597	Temp. North	Temperate	Estuary NAEC
TN6	GOS007	0.510	Temp. North	Temperate	Coast NAEC
TO1	GOS015	1.274	Open ocean	Tropical	Gulf stream
TO2	GOS016	1.271	Open ocean	Tropical	Gulf stream
TO3	GOS017	2.576	Open ocean	Tropical	Gulf stream
TO4	GOS018	1.427	Open ocean	Tropical	Gulf stream
TO5	GOS019	1.353	Open ocean	Tropical	Caribbean
TO6	GOS023	1.331	Open ocean	Tropical	Trop. East Pacific
TO7	GOS026	1.027	Open ocean	Tropical	Galapagos
TS1	GOS008	1.297	Temp. South	Temperate	Coast NAEC
TS2	GOS009	0.793	Temp. South	Temperate	Coast NAEC
TS3	GOS010	0.783	Temp. South	Temperate	Coast NAEC
TS4	GOS013	1.380	Temp. South	Temperate	Coast NAEC

### 2.2 Dissimilarity measures

For our assessment, we chose two popular dissimilarity measures used in metagenomics samples comparison: the abundance-based Bray-Curtis dissimilarity measure (Bray and Curtis, 1957) and the presence-based Jaccard distance.

#### 2.2.1 Bray-Curtis dissimilarity

One of the most frequently adopted indices for β -diversity in ecology is the Bray-Curtis dissimilarity index (BC). In its original definition, it measures how many individuals from one of the two samples cannot be coupled with an individual of the same species from the other sample, and it normalizes such a quantity by the mean number of individuals per sample.

Let $S^{*}$ be the number of species in an environment E , and $N_{X,i}$ be the number of individuals of the i -th species in a sample X of E , i.e., $N_{X}^{*}=\sum_{i=1}^{S^{*}}N_{X,i}$ is the number of individuals in the sample X . Given two metagenomic samples A and B , we define:

$$BC\;\left(A,B\right)=1-2\frac{\sum_{i=1}^{S^{*}}min\left(N_{A,i},N_{B,i}\right)}{N_{A}^{*}+N_{B}^{*}}$$

In (Dubinkina et al., 2016) the BC dissimilarity measures is re-defined in terms of k -mers, thus providing a reference-free version. Given two sets A and B of metagenomics reads, let $N_{A}\left(w_{i}\right)$ be the number of occurrences of the k -mer $w_{i}$ in A and $N_{B}\left(w_{i}\right)$ the number of occurrences of the k -mer $w_{i}$ in B . Then, the Bray-Curtis dissimilarity measure between A and B is defined as:

$$BC\;\left(A,B,k\right)=1-2\frac{\sum_{i=1}^{4^{k}}min\left(N_{A}\left(w_{i}\right),N_{B}\left(w_{i}\right)\right)}{\sum_{i=1}^{4^{k}}\left(N_{A}\left(w_{i}\right)+N_{B}\left(w_{i}\right)\right)}$$

Note that, in ecology, normalization of the number of individuals can be done on the basis of the area (e.g., square meters) in which the individuals are counted. In case of k -mers, normalizing vectors before computing Bray-Curtis dissimilarity would actually lead to Manhattan distance computation. However, as explained in Benoit et al. (2016), the equation involves marginal (or dataset specific) terms (i.e., $\sum_{w\in \Sigma^{k}}N_{A}\left(w\right)$ is the total amount of k -mers in dataset A ) acting as normalizing constants and crossed terms that capture the (dis)similarity between datasets (i.e., $\sum_{w\in \Sigma^{k}}\min\left(N_{A}\left(w\right),N_{B}\left(w\right)\right)$ is the total amount of k -mers in the intersection of the datasets A and B). Marginal and crossed terms are then combined to compute the final distance.

#### 2.2.2 Jaccard distance

In the context of metagenomics, the Jaccard distance compares the number of unshared species to the total number of species in the combined samples. Therefore, it provides a real value in [0,1] that can be interpreted as the probability of randomly selecting an unshared species among all species detected in the samples. In other words, the closer the samples, the smaller the Jaccard distance.

Let $S_{A}=\left\{i\;:\;N_{A,i}>0\right\}$ be the set of species in the metagenomic sample A and $S_{B}=\left\{i\;:\;N_{B,i}>0\right\}$ be the set of species in the metagenomic sample B , the Jaccard similarity index is defined as follows:

$$J\left(A,B\right)=\frac{\left|S_{A}\cap S_{B}\right|}{\left|S_{A}\cup S_{B}\right|}$$

The Jaccard distance is consequently defined as:

$$d_{J}\left(A,B\right)=1-J\left(A,B\right)$$

In the k -mer-based version of the Jaccard distance, the sets A and B are indeed the sets of k -mers extracted from all reads of each sample:

$$J\left(A,B,k\right)=\frac{\left|w_{i}\in \Sigma^{k}\text{ such that: }N_{A}\left(w_{i}\right)\neq 0\wedge N_{B}\left(w_{i}\right)\neq 0\right|}{\left|w_{i}\in \Sigma^{k}\text{ such that: }N_{A}\left(w_{i}\right)\neq 0\vee N_{B}\left(w_{i}\right)\neq 0\right|}$$

#### 2.2.3 Computation of the dissimilarity matrices

For the computation of the dissimilarity matrices between the samples in our datasets, we used different strategies depending on whether the ground truth was available or not.

For the collection of simulated metagenomes from the CAMI initiative, we computed their metagenomic dissimilarity matrices using true species abundances derived from mapping each read identifier to the NCBI taxonomy database.

For the collections of real metagenomics samples, for which true species distributions are not available, we used a taxonomic classifier to estimate them. First, the reads of each sample were classified with Kraken2 ((Wood et al., 2019)), exploiting the standard reference database that includes bacterial, archaea, viral, and plasmid sequences. The estimated species abundances were then obtained using Bracken ((Lu et al., 2017)) with default parameters. We then used such abundance vectors to get dissimilarity matrices through the distance functions available from the R library ecodist, and considered them as a proxy of the ground truth for real datasets.

For all the collections, we then ran Simka (Benoit et al., 2016) to get the exact k -mer-based dissimilarity matrices computed over all available k -mers (except singletons) in each metagenomics sample dataset, for different lengths of k -mers. We underline that the purpose of this study is not to benchmark different tools for the comparison of metagenomic samples, but to assess the power of reference-free k -mer-based definitions of Bray-Curtis and Jaccard distances. For this reason, it is not relevant which tool we use to compute the distances based on k -mers; in principle, we could have even used a naive algorithm. We chose the Simka tool because it is well documented and easy to use.

### 2.3 Statistical measures of correlation

To compare two dissimilarity matrices, we applied a Mantel test, using the scikit-bio function Mantel with 999 permutations, to the distance matrices computed with the k -mer-based reference-free and reference-based approaches (or with the ground-truth in case of simulated datasets). We used Pearson and Spearman correlations, since both of them carry relevant information. In fact, a high Pearson correlation indicates a strong linear relationship between two variables. Consequently, it is reasonable to expect that two highly correlated dissimilarity matrices in the Pearson’s sense would have similar clustering at higher levels of the clustering tree. A high Spearman correlation, on the other hand, being a rank correlation and thus indicating mutual monotonicity of two variables, is more likely to produce similar clustering at a low level of the tree. However, the main reason for considering Spearman correlation is that dissimilarities based on k mer and taxonomy are not linearly related in principle, yet we are interested in their discriminative power, which is captured by ranking dissimilarities.

## 3 Results and discussion

In this section, we report results on how well the investigated k -mer-based β -diversity indices, computed among metagenomes samples, correlate with their respective reference-based indices or with the ground-truth when available. We ran such analysis for both Bray-Curtis and Jaccard indices, and for different, increasing, k -mer lengths. Results with respect to both Spearman and Pearson correlations are reported and discussed.

### 3.1 Experiments on Bray-Curtis dissimilarity

We tested the Bray-Curtis dissimilarity index on a simulated Human dataset from CAMI and on two real datasets including samples from the GOS expedition and the Human Microbiome Project.

#### 3.1.1 BC on a simulated human dataset

We first analyze the behavior of Bray-Curtis dissimilarity based on k -mer compared to the ground truth in the simulated human dataset, and report the results in Table 4.

**TABLE 4:** Correlations between k -mer-based Bray-Curtis dissimilarities computed with the reference-free approach and Bray-Curtis dissimilarites computed with the reference-based Bracken, with respect to the true dissimilarity in the CAMI simulated dataset. The reported values have been obtained with a Mantel test with 999 permutations.

k	Pearson’s	Pearson	Spearman’s	Spearman
	Correlation	p-value	Correlation	p-value
16	0.987	0.001	0.849	0.001
21	0.994	0.001	0.987	0.001
26	0.994	0.001	0.987	0.001
31	0.994	0.001	0.989	0.001
Bracken	0.990	0.001	0.986	0.001

Both Pearson and Spearman correlations reveal that, for k -mer lengths k∈{21,26,31} , the correlation of the estimated measures of dissimilarity, with respect to the ground truth, is high and comparable (actually slightly higher) to the one of Bracken reference-based approach. Moreover, they are also extremely high and stable for large values of k , vouching for the validity of sequence-composition reference-free methods for measuring metagenomics diversity.

**FIGURE 1:**
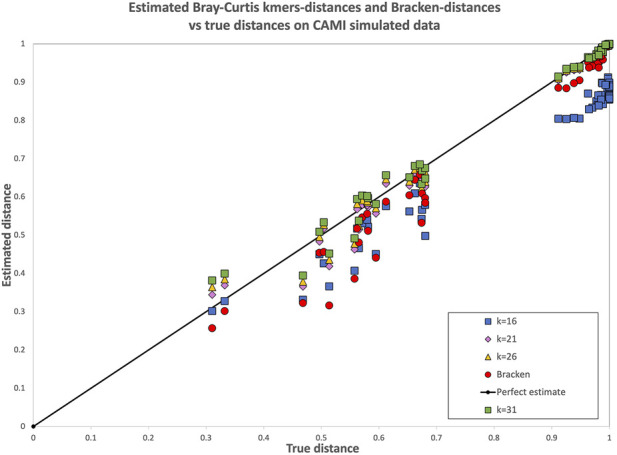
Bray-Curtis dissimilarity computed with the reference-free k -mer approach and with Bracken for all pairs of samples in the CAMI simulated dataset. Each point represents the dissimilarity between two samples: the x-coordinate is the real dissimilarity value, while the y-coordinate is the one obtained with either the reference free k -mer approach (for various k) or with the abundances estimated with Bracken. For k≥21 the reference-free approach has not only a similar trend, but the computed distances are all very close to the diagonal, i.e., to the ground truth. In this dataset Bracken-based distances underestimate the distance for pairs of samples of medium dissimilarity [0.5–0.6], while they are closer to the ground truth for pairs of very different samples (distance ≥0.9 ).

However, when 16-mers were used, a decay is evident. Looking in detail at the distances between each pair of samples in the dataset (see Figure 1), it is evident that for k=16 the drop in correlation is caused by pairs of highly dissimilar metagenomes (0.9≤BC≤1) for which the 16-mer-based BC dissimilarity is estimated in the range 0.8≤BC≤0.9 . This suggests, indeed, that 16-mers are too short for a metagenomics comparison at the species level in this collection.

Bracken-based BC dissimilarity also correlates quite well with truth in the simulated CAMI collection. To complement Figure 1, in which some values for k≥21 overlap and are different to distinguish, we report in Table 5 the mean and variance of the absolute residuals with respect to the ground truth for all the approaches. The data confirms that k=16 presents the highest error, while for k≥21 all reference-free approaches have a similar mean error, slightly better than the one of Bracken.

**TABLE 5:** The mean and variance of absolute residuals among al pairs of computed BC distances in the CAMI dataset for each tested method. For variance we have reported 4 decimals, to highlight that there is one order of magnitude between the variance of Bracken and k=16 and the variance for k≥21 .

	Bracken	k=16	k=21	k=26	k=31
Mean	0.0310	0.1080	0.0130	0.0120	0.0120
Var	0.0019	0.0013	0.0005	0.0004	0.0003

**FIGURE 2:**
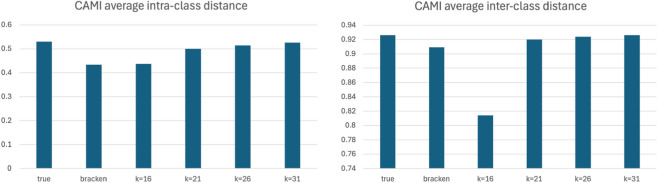
Average intra-sample dissimilarity (left) and inter-sample dissimilarity (right) among the sample sites of the CAMI dataset. For values of k≥21 the reference-free approach is always closer to the ground truth than the reference-based Bracken approach. The low values estimated between pairs of distant samples for k=16 can be explained looking at the top-right corner of Figure 1 where we can see a cluster of blue squares that is clearly below the diagonal, while for other values of k and for Bracken the points are much closer to the diagonal.

To deeply analyze the ability of the tested methods to relate to a known truth, we also computed the average distances between samples within the same environment and between different environments. The results are reported in Figure 2. Again, it is evident that reference-free BC dissimilarity for k≥21 relates to the ground truth very well, and slightly better than Bracken. For k=16 it is evident that are the most dissimilar pairs of samples the ones responsible for the drop in performances for this k value.

#### 3.1.2 Bray-Curtis on the GOS dataset

The collection from the GOS expedition is characterized by a much higher number of smaller samples than the CAMI simulated dataset. For this dataset, we do not have the ground-truth available, therefore we computed the correlation scores with respect to the distances obtained with the reference-based approach Bracken. In addition, in our evaluation, we also looked at the classification and clustering analysis carried out by (Rusch et al., 2007) at two levels of granularity, and compared it with the clustering obtained with the reference-free k -mer-based BC dissimilarity and with the Bracken approach.

In GOS metagenome samples the highest correlations between reference-free and reference-based indices are obtained using 14mers. While the correlations are still quite high (0.888 for Pearson and 0.859 for Spearman) it is lower than the one reported for the CAMI simulated dataset (see Table 6). The Pearson correlation tends to decrease with higher values of k (0.667 for k=31 ), while the corresponding Spearman values are stable and around 0.8 (0.841 for k=31 ), showing the relative rank is maintained even if the actual values computed with the reference-free approach do not necessary follow a very strong linear correlation with those computed by Bracken.

**TABLE 6:** Correlations between Bray-Curtis dissimilarities computed with the reference-free k -mer-based approach and those Bracken-based in GOS dataset. The reported values have been obtained with a Mantel test with 999 permutations.

k	Pearson’s	Pearson	Spearman’s	Spearman
	Correlation	p-value	Correlation	p-value
12	0.802	0.001	0.774	0.001
14	0.888	0.001	0.859	0.001
16	0.858	0.001	0.857	0.001
21	0.720	0.001	0.844	0.001
26	0.688	0.001	0.837	0.001
31	0.667	0.001	0.831	0.001

**FIGURE 3:**
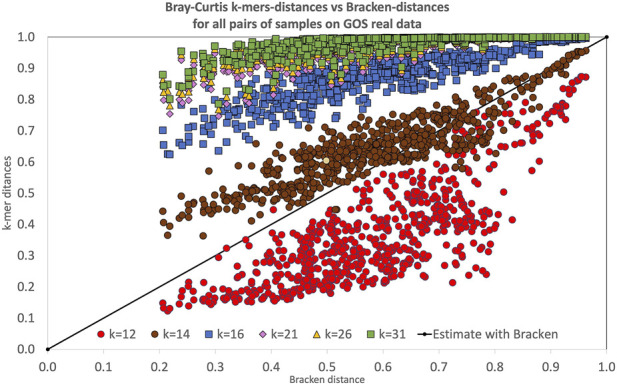
Bray-Curtis dissimilarity computed with the reference-free k -mer approach, for several values of k for all pairs of samples in the GOS real dataset. Each point represents the dissimilarity between two samples: the x -coordinate is the dissimilarity computed with Bracken, while the y -coordinate is the one obtained with the reference free k -mer approach (for various k ). A perfect agreement between the two approaches would place points along the diagonal.

Looking in detail at the distribution of pairs of distance among the samples in the dataset, as reported in Figure 3, we observe that for k=12 the estimated dissimilarity is constantly lower than the one estimated with Bracken. Moreover, the values are spread over a large interval. On the other hand, for larger values of k the trend of the values appears to be more linear, although higher dissimilarity values are estimated with respect to Bracken. Even visually, the value of k that show the closest distance estimates to those of Bracken is k=14 , in line with the computed values of correlations. The figure also shows that for k≥14 the k -mer based approaches tend to over-estimate the distance between closed samples with respect to the one computed with Bracken. This is probably due to the fact that increasing values of k lead to a larger number of different k -mers, thus resulting in a larger difference than the one actually present between the compared samples. Since the values for k≥21 are all very similar and difficult to distinguish, we have calculated for this experiment also the mean and variance of absolute residuals, as reported in Table 7.

**TABLE 7:** The mean and variance of absolute residuals among al pairs of computed BC distances in the GOS dataset for each tested method. For k=14 we have the smallest average absolute residuals and the least variance with respect to the distances computed with Bracken.

k	12	14	16	21	26	31
Mean	0.216	0.076	0.282	0.376	0.384	0.388
Var	0.010	0.003	0.011	0.017	0.018	0.019

**FIGURE 4:**
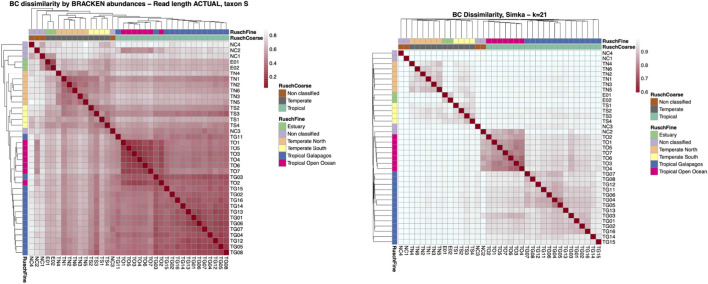
Comparison between the heatmaps of Bray-Curtis dissimilarities in the GOS dataset computed with Bracken (left) and with the k -mer-based BC with k=21 (right). By looking at the reconstructed phylogeny on the top and left side of the heatmaps, it is possible to note that the k -mer-based BC, with k=21 correctly separate the Tropical subsets (pink and blue samples), while Bracken reports some misplacement.

Interestingly, the best clustering of metagenomes according to the classification proposed in ((Rusch et al., 2007)) is obtained with k=21 . Figure 4 shows the details of the clustering. In particular, Figure 4 (left) shows the heatmap obtained with Bracken, while Figure 4 (right) shows the one obtained with the reference-free k -mer-based approach for k=21 . We observe that, for opposite reasons depending on the general under- and over-estimation of the dissimilarities, in both heatmaps it is not easy to visually distinguish the clusters, although the main clusters are evident as squares along the diagonal in both cases. However, by looking at the phylogenies built with the computed distances, we can see that k=21 correctly groups Tropical Open Ocean and Tropical Galapagos as in (Rusch et al., 2007), while Bracken report a misplacement. Thus, although the best correlation to Bracken is obtained for k=14 , the results that are closer to an independent analysis are obtained with the larger value of k=21 .

#### 3.1.3 Bray-Curtis on a human microbiome project dataset

**FIGURE 5:**
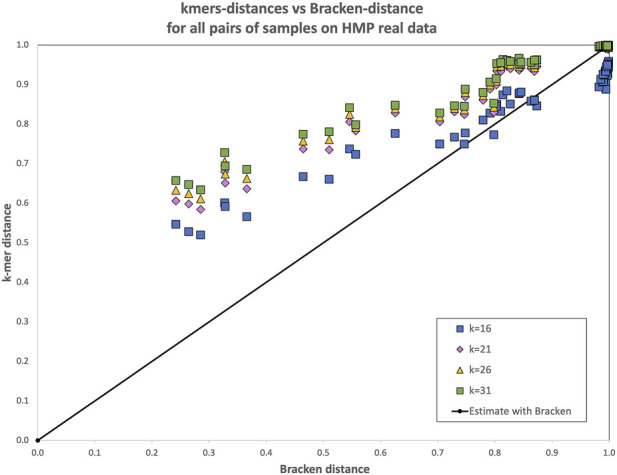
Bray-Curtis dissimilarity computed with the reference-free k -mer approach, for several values of k for all pairs of samples in the HMP real dataset. Each point represents the dissimilarity between two samples: the x -coordinate is the dissimilarity computed with Bracken, while the y -coordinate is the one obtained with the reference free k -mer approach (for various k ). A perfect agreement between the two approaches would place points along the diagonal.

In the real HMP dataset Pearson correlations stick to our previous observations for the HMP simulated dataset, but for a slightly clearer differentiation between indices based on different k-mer lengths, as shown in Table 8. We recall that here we are not comparing against the ground truth, which is not available, but against the results provided by Bracken abundance estimate. Both the tested correlations are above 90%, with the Pearson correlation stable at a value of 98% with small fluctuations depending on the value of k . These fluctuations are amplified with the Spearman correlation, although still quite high (between 90% and 96%). The drop in Spearman correlation can be explained by the presence of cluster of points that are very close to each other (see Figure 5): their relative position in the ranking might be affected even if the k -mer distances are close to the one predicted by Bracken.

**TABLE 8:** Correlations between k -mer-based reference-free Bray-curtis dissimilarities and those Bracken-based in HMP dataset. The reported values have been obtained with a Mantel test with 999 permutations.

k	Pearson’s	Pearson	Spearman’s	Spearman
	correlation	p-value	correlation	p-value
16	0.983	0.001	0.964	0.001
21	0.985	0.001	0.906	0.001
26	0.983	0.001	0.913	0.001
31	0.981	0.001	0.922	0.001

Despite these differences, the correlation values are high for all values of k we tested, as confirmed by the mean of the absolute residuals in Table 9. The table also show that k=16 holds the smallest variance. The higher variance observed for k≥21 is explained by the presence of larger differences between pairs of samples with low dissimilarity than the ones observed between very dissimilar samples (see Figure 5).

**TABLE 9:** The mean and variance of absolute residuals among al pairs of computed BC distances in the HMP dataset for each tested method. We can notice that there is less variability in the absolute difference between the distances of Bracken and k=16 , than with larger values of k .

k	16	21	26	31
Mean	0.079	0.079	0.085	0.091
Var	0.005	0.011	0.013	0.014

**FIGURE 6:**
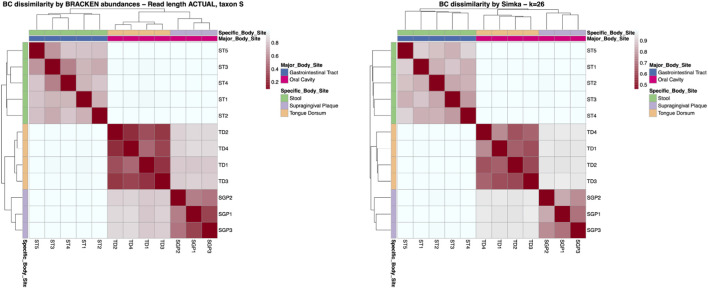
Comparison between the heatmaps of BC dissimilarities in the HMP real dataset computed with Bracken (left) and with the reference-free k -mer-based approach for k=26 . Both methods clearly distinguish among sample sites.

This is also reflected in the same ability to distinguish among samples taken from different sites, as can be seen from the heatmaps in Figure 6 that report the comparison between the heatmap obtained with Bracken and the one obtained for k=26 (for k=16,21,31 we obtained similar results).

### 3.2 Experiments on the jaccard distance

We repeated the former analysis to study the behavior of the Jaccard distance on varying k -mer lengths. From our analysis, this index appears to be less linearly correlated in its k -mer version compared to the true taxonomy in the CAMI dataset (see Table 10). In contrast, the Spearman correlation between the true and k mer Jaccard distances is high and even higher than that obtained with the reference-based Bracken Jaccard index. This suggests that despite the fact that the estimate of the distances might not be perfectly linearly correlated, their ranking. i.e., the relative order defined by the similarity among samples, is more preserved.

**TABLE 10:** Correlations between k -mer-based Jaccard distance computed with a reference-free k -mer-based approach and Jaccard distance computed with Bracken, with respect to the true distance on the CAMI simulated dataset. The reported values have been obtained with a Mantel test with 999 permutations.

k	Pearson’s	Pearson	Spearman’s	Spearman
	correlation	p-value	correlation	p-value
16	0.867	0.001	0.795	0.001
21	0.842	0.001	0.937	0.001
26	0.827	0.001	0.932	0.001
31	0.827	0.001	0.934	0.001
Bracken	0.924	0.001	0.926	0.001

With both real datasets (see Tables 11, 12) the drop in correlation is also perceivable when ranking is involved. In HMP samples the Pearson’s correlation is stable across values of k while among GOS samples shorter k -mers (12≤k≤16) produce Jaccard distances that are more correlated to the reference-based distance than those computed with higher values of k . In terms of relative order, the Spearman correlation peaks on relative short k -mers (12–16 bp), with k=14 showing the best correlation.

**TABLE 11:** Correlations between k -mer-based Jaccard distance and those Bracken-based in GOS dataset. The reported values have been obtained with a Mantel test with 999 permutations.

k	Pearson’s	Pearson	Spearman’s	Spearman
	correlation	p-value	correlation	p-value
12	0.773	0.001	0.777	0.001
14	0.882	0.001	0.893	0.001
16	0.649	0.001	0.666	0.001
21	0.431	0.001	0.487	0.001
26	0.399	0.001	0.473	0.001
31	0.379	0.001	0.466	0.001

**TABLE 12:** Correlations between k -mer-based Jaccard distance and those Bracken-based in HMP dataset. The reported values have been obtained with a Mantel test with 999 permutations.

k	Pearson’s	Pearson	Spearman’s	Spearman
	correlation	p-value	correlation	p-value
16	0.768	0.001	0.728	0.001
21	0.778	0.001	0.660	0.001
26	0.778	0.001	0.672	0.001
31	0.776	0.001	0.691	0.001

In environmental metagenomics, Jaccard distance often performs worse than Bray-Curtis because environmental samples are usually dominated by a few highly abundant organisms, and the presence of low-abundance organisms is highly variable due to possible errors and/or limitations in sampling technologies. Thus, in environmental samples, the structural difference is often driven by massive shifts in dominant taxa, not just by the presence of rare species. For this reason, abundance-based dissimilarities, such as Bray-Curtis can better intercept the (dis)similarity between samples. This clearly still holds when the distances are based on k -mer abundance rather than presence, because the variations in species abundance will affect the k -mers counts, but not the presence, for dominant species.

In our experiments, we can see that, in the simulated CAMI datasets, the difference between the correlation computed using Jaccard and the values computed with Bray-Curtis is between 10% and 15%, with small fluctuations due to the value of k when we measure linear correlation. Instead, when the correlation is measured with respect to the ranking the difference is only about 5%. This dataset is characterized by reads that have all the same length and all the datasets have the same number of reads, thus the total number of kmer is the same for all the datasets. The number of distinct kmers evidently change (as Jaccard distance is less correlated with the ground truth) but it remains within a reasonable range.

In real datasets, the number of reads can vary from sample to sample, and within each sample the read length can also vary. This can impact the values of the computed dissimilarity. In our experiments, the HMP datasets are characterized by a very similar number of reads and reads length. However, we observe a decrease of 20% for the Pearson correlation and of 25%–30% for the Spearman correlation with respect to the correlations computed with the BC dissimilarity. The larger difference in Spearman correlation can be explained by small differences in the distance values that do not affect linear relationship, but nevertheless lead to differences in ranking.

The GOS dataset instead presents a high variability in terms of reads in each sample, and read length. Both correlations clearly drop when k increases. This can be explained by observing that longer k -mers have less occurrences. If they also belong to a rare species it is possible that they go undetected because the corresponding reads are not sequenced, or not entirely sequenced or sequenced with large noise and then dropped. In BC the abundance of the species can partially compensate, while in Jaccard the k -mer will simply be missing.

Summarizing, Bray-Curtis tells us how similar samples are in terms of community structure, and receive more contribution from differences between dominant microbes. Jaccard tells us if the samples are similar in terms of compositional list, e.g., if they share the same list of species, regardless of count.

If Bray-Curtis gives better clustering, as it is the case of our experiments, it means that the difference between samples in the studied environments are driven by the dominant species’ relative abundances rather than caused by a complete turnover of species presence.

Jaccard distance should therefore be avoided when there are large differences in the number of reads or read lengths across samples, when the aim of the study is to estimate the difference between functional components of the community that are more affected by dominant species, and when the data is sparse and technology errors could make a species to be undetected.

## 4 Conclusion

The contribution of this work is an experimental validation of a widely used, but not properly verified, assumption in metagenomic analysis: that reference-free k -mer–based dissimilarity measures are able to capture differences between samples for large values of k . We tested this hypothesis at a species level across a wide range of k values, using both linear and rank-based correlation metrics, on a simulated dataset with respect to the known ground truth, and on two real datasets from different kind of environments, the oceans and the human body, with respect to the dissimilarity computed by a reference-based approach (Bracken).

Within the framework of our experimental settings, the results on simulated datasets showed strong and robust correlation with the ground truth up to k=31 . Moreover, in this context, we noted that reference-free k -mer approaches tend to slightly overestimate the real distance for k≥21 , while a reference-based approach such as Bracken tends to underestimate it. For the analysis on the real datasets considered in this work, the correlation was computed with respect to the distances obtained with Bracken, used as a proxy for the ground truth, providing empirical evidence that, at the species level, reference-free k -mer–based distances can serve as reliable alternative when reference-based approaches are computationally impractical.

Moreover, with our analysis we give hints of which values of k should be used on several different settings. In particular, the reference-free k -mer-based version of the Bray-Curtis dissimilarity measure is well correlated and stable with respect to the real samples taxonomy for values 16≤k≤31 in the HMP dataset characterized by short reads. For the GOS dataset, characterized by a smaller number of reads with higher variability in number and length, values 14≤k≤16 showed high correlation both in terms of Pearson and Spearman. In terms of distance to the actual values predicted with Bracken, k=14 was the value that showed the better approximation and smaller variance among pairs of samples.

For a presence-based distance, such as Jaccard, the correlation with the ground truth in simulated datasets is still very strong, although less evident with Pearson than with Spearman for k≥21 . A similar behavior has been reported for the real HMP dataset, where the Pearson is stable and above 0.76 for all the tested values of k , while the Spearman correlation shows some fluctuations between 0.66 and 0.72 for the different k . In real GOS dataset, where samples are characterized by a relative smaller number of reads $\left(10^{6}\right)$ and with more variability in reads length, the Pearson correlation drop for large values of k , but it is still preserved for smaller values in the range (12≤k≤14) , both in terms of Pearson and Spearman.

We believe this validation gives useful insights for both method selection and experimental design in metagenomic studies, particularly in large-scale or less studied environments with reference-poor settings.

## Data Availability

The datasets analyzed for this study can be downloaded from the respective repositories as explained in the Material and Methods section. We have made available a script to download the GOS dataset and the links to the software in a public reposotory: https://github.com/PizziLab/MetagenomicsKmerComparison.

## Footnotes

1 As at the time of submission of the manuscript this link is not working, the datasets can alternatively be downloaded from the Sequence Read Archive, https://www.ncbi.nlm.nih.gov/sra, searching the accession numbers in Table2
